# A virtual reality study investigating the train illusion

**DOI:** 10.1098/rsos.221622

**Published:** 2023-04-12

**Authors:** Lars Kooijman, Houshyar Asadi, Shady Mohamed, Saeid Nahavandi

**Affiliations:** ^1^ Institute for Intelligent Systems Research and Innovation, Deakin University, Geelong, Victoria, Australia; ^2^ Harvard Paulson School of Engineering and Applied Sciences, Harvard University, Allston, MA 02134, USA

**Keywords:** vection intensity, qualitative survey, mixed methods, physiological responses

## Abstract

The feeling of self-movement that occurs in the absence of physical motion is often referred to as vection, which is commonly exemplified using the train illusion analogy (TIA). Limited research exists on whether the TIA accurately exemplifies the experience of vection in virtual environments (VEs). Few studies complemented their vection research with participants' qualitative feedback or by recording physiological responses, and most studies used stimuli that contextually differed from the TIA. We investigated whether vection is experienced differently in a VE replicating the TIA compared to a VE depicting optic flow by recording subjective and physiological responses. Additionally, we explored participants' experience through an open question survey. We expected the TIA environment to induce enhanced vection compared to the optic flow environment. Twenty-nine participants were visually and audibly immersed in VEs that either depicted optic flow or replicated the TIA. Results showed optic flow elicited more compelling vection than the TIA environment and no consistent physiological correlates to vection were identified. The post-experiment survey revealed discrepancies between participants' quantitative and qualitative feedback. Although the dynamic content may outweigh the ecological relevance of the stimuli, it was concluded that more qualitative research is needed to understand participants' vection experience in VEs.

## 1. Introduction

The subjective experience of self-motion in the absence of physical motion, also known as vection, can occur in response to strong visual stimuli. Although vection has been exemplified as a visual illusion, it can be elicited, and modulated, through a variety of senses, such as auditory [1,2], tactile [3,4] and biomechanical stimulation [5,6]. Furthermore, it has been shown that (synchronized) multisensory feedback can increase vection compared to unisensory feedback (e.g. [4,7–9]).

There are several reasons why the scientific investigation of vection is important. First, vection research could improve our understanding of how humans perform functionally significant daily tasks as we gain understanding how (illusory) self-motion information is processed by our sensory system. Specifically, Palmisano *et al*. [10] suggested that vection could play a role when we make judgements (i.e. possible functional role no. 1) of self-motion or when we control (i.e. possible functional role no. 2) self-motion. To spatially navigate and orient ourselves, we must integrate sensory cues in order to infer and control our movement [11,12]. Riecke *et al*. [13] identified that vection facilitates perspective switching, which is used in spatial orientation, highlighting the possible functional role of vection. Second, prior research found vection and presence to be correlated [14], which suggests that the experience of vection is desirable for virtual reality (VR) applications. Therefore, vection research could contribute to enhancing the fidelity of VR applications [15]. Finally, mixed findings have been reported in the literature [16,17] regarding the relationship between vection and visually induced motion sickness (VIMS). Vection and VIMS have a rather complex relationship (see [18]) and further research is required to understand this relationship.

A variety of conceptual and methodological concerns are present in the current vection literature (e.g. [2,3,10,19,20]). In addition to the lack of a consistent definition of vection, as noted by Palmisano *et al*. [10], both (i) explaining the concept of vection to participants, and (ii) identifying physiological correlates to vection are among the most pressing issues.

### 1.1. Explaining vection

Vection is often exemplified by the train illusion. Authors use the train illusion to describe the concept of vection to either (i) the readership (e.g. [18–41]), or (ii) to participants (e.g. [34,42–46]). The train illusion has been described to occur when a person is seated in a stationary train, and a stationary train adjacent to the person starts moving. Subsequently, the person in the stationary train perceives that they are moving in the opposite direction to that of the adjacent train. Objectively, the movement of the adjacent train is considered ‘scene motion' or ‘object motion' and thus should be interpreted by the observer as motion information that is relative to a stationary observer. However, one possible explanation why vection occurs when one is seated on a train could be that the optic flow induced by the adjacent train is unconsciously, and erroneously, interpreted by the observer as the movement of the observer relative to the environment, which is further modulated by the potential expectation of the observer that their train is moving again soon. There are several (historical) anecdotal accounts of illusions surrounding trains, which may have formed the foundation for the use of the train illusion to explain vection in scientific research.

Von Helmholtz [47] used the term ‘giddiness' (*Schwindel* [47, p. 602]; translated from original German text) to describe the illusory motion of objects and detailed the occurrence of this phenomenon by describing someone who is sitting on a moving train and switches their focus from looking at objects close to the track outside of the train to the floor inside of the train carriage. Subsequently, the floor of the carriage appears to move in the same direction as the train. Von Helmholz suggested that the illusory perception arises owing to an after-effect from ‘impulses of the will' (*Willensimpulse* [47, p. 603]; translated from original German text): the observer had to make quick eye movements while looking at objects close to the track and having got accustomed to them, these eye movements are retained when the observer tries to focus on stationary objects. Thus, stationary objects appear to be moving. Similarly, Leake [48] described the occurrence of illusory motion perception, which occurs when one is located at the end of the train. If the observer watches the landscape receding while the train is moving, then when the train stops moving, the landscape appears to be moving towards the observer. Leake hypothesized that the occurrence of these illusory perceptions is owing to persistent rhythmic accommodative activities. Although both accounts detail illusory (self) motion perception elicited by the movement of a train, they require the observer to perceive movement prior to the occurrence of the illusion. As such, William James' description is perhaps the most accurate description of the train illusion. James [49] denoted that: ‘*… [t]here is an illusion of movement of the opposite sort with which everyone is familiar at railway stations. … when another train … fills the entire window and … begins to glide away we judge that it is our train which is moving and that the other train is still*’ [49, pp. 90–91]. Similarly Fischer & Kornmüller introduced readers to their research on movement perceptions by exemplifying ‘well-known phenomena or impressions' (i.e. ‘*eindrucksvolle Ercheinungen*', [50, p. 273]; translated from original German text), one of which was the train illusion that was described as: ‘*…if a train on the adjacent track starts moving one often thinks their own train drives off in the opposite direction whereas the adjacent train appears motionless*.' [50, p. 273; translated from the original German text].

Presumably, these anecdotal accounts form the basis of the train illusion example, which pervades scientific literature on vection research. However, to the best of our knowledge, only a few studies [32,34,38,51,52] have employed contextually similar (i.e. train-related) cues to investigate vection whereas most studies use stimuli which vary contextually from the description of train illusion. For example, visual stimuli in the form of optic flow patterns are commonly employed, in which participants perceive an array of approaching or receding points or squares (e.g. [35,53–55]) which are aimed at eliciting *linear vection*. Alternatively, researchers have used vertical gratings (e.g. [7,44]), such as those presented in optokinetic drums, to elicit c*ircular vection*. In addition to abstract visual cues, more realistic visual stimuli have been used to elicit vection, where, for example, participants perceive that they are sandboarding down a dune [29], diving in the ocean [25] or riding a bicycle [23].

Sequentially, a variety of auditory cues have been used; Väljamäe *et al*. [56] presented 23 blindfolded participants with approaching or receding auditory landmarks reflecting the engine of a bus or a barking dog to induce *linear vection*. Similarly, Keshavarz *et al*. [7] aimed to elicit *circular vection* by presenting 20 blindfolded participants with the sound of a church bell played through an array of speakers to replicate auditory rotation. Tinga *et al*. [45] investigated whether vibrational stimulation around the circumference of participants' waist could elicit or modulate vection whereas Kruijff *et al*. [8] presented vibrational cues to participants’ feet to investigate whether vection induced by virtual locomotion was enhanced. Biomechanical cues were used in the study by Riecke *et al*. [6] where 19 blindfolded participants performed side-stepping motions on a circular treadmill to induce *circular vection* while participants were sitting stationary on a hanging chair. Fourteen blindfolded participants in the study by Bles *et al*. [57] sat in a chair and performed hand-over-hand movements on a moving board in front of them, which elicited a sensation of self-motion to the left or right in over one-third of the participants.

In summary, there appears to be a disassociation between the context of the train illusion analogy (TIA) and the stimuli used in the research, which may alter participants' response behaviour to vection-inducing stimuli. Such a disassociation was noted by the 12 participants recruited in the study conducted by Soave *et al*. [38]; participants indicated that the train illusion did not reflect their experience of vection. Nonetheless, little research has been conducted to assess whether the description of the train illusion is an appropriate exemplification of the feeling of self-motion which participants appear to have in response to (abstract) stimuli in VR. Conversely put, there seems to be a lack of understanding as to whether the train illusion is an appropriate descriptor for the subjective experience of self-motion in virtual environments (VEs). Having an appropriate descriptor of vection is important because research has shown that vection can be influenced by top-down (cognitive) factors, such as task instructions [58], the ecological relevance of the stimulus [14,59], attentional load [60,61] and expectations and/or beliefs about the possibility of physical self-motion occurring [23,36].

### 1.2. Physiological correlates to vection

Although various researchers have investigated neurophysiological (e.g. [27,62,63], see [19] for a review), postural (e.g. [64–66]) and behavioural (e.g. [67]) correlates to vection, there appears to be a lack of studies investigating responses from the autonomic nervous system that might correlate to participants' experience of vection. By contrast, psychophysiological measures of cybersickness (CS), VIMS and presence are employed more commonly, where researchers have identified physiological indices associated with the (de)activation of the (para)sympathetic nervous system that correlate to participants' experience of CS, VIMS or presence (see [68–70] for reviews). Opposed to the wide range of studies investigating the psychophysiological response to CS, VIMS and presence, to the best of our knowledge, only two studies exist which investigated a physiological index, or physiological indices, of the autonomic system in participants that were experiencing vection. Aoki *et al*. [71] recorded electrocardiograms (ECGs), arterial blood pressure, electrodermal activity (EDA) and respiration rate of participants seated in a motion platform. Ten male participants were subjected to physical tilts and optokinetic stimulation aimed to elicit vection and indicated the onset and offset of their vection experience by pressing a button. The authors found two different responses; participants either had (i) an autonomic suppressor, or (ii) an autonomic activation response to vection. However, no data was collected on the intensity or convincingness of their vection experience to correlate the subjective dimensions of vection to the physiological responses. Participants in the study by Ihaya *et al*. [72] were presented with optic flow patterns while they performed a clapping task and had their pupillary activity recorded. The authors in this study found that (i) participants' pupil sizes increased over the duration of exposure, and (ii) that larger dilations were present for faster optic flow speeds compared to slower speeds. Of note, participants' ability to experience vection was determined *before* the experimental trials wherein participants performed a clapping task, but no explicit (subjective) measure of vection was reported that verified whether participants indeed experienced vection *while* they performed the clapping task. Thus, these dilative effects may have been owing to the physical effort of clapping (e.g. [73,74], on the effect of physical activity on pupil size). In summary, there appears to be limited and conflicting evidence regarding the activity of the autonomic nervous system in response to vection.

### 1.3. Goals of current study

In the current study, we had a set of goals that can be subdivided into four main components, namely (i) ecological relevance, (ii) sensory modality, (iii) physiological responses, and (iv) understanding participants' vection experience. The goals and hypothesis are detailed for each component in the following sections.

#### 1.3.1. Ecological relevance

We aimed to compare whether vection is experienced differently in a VE of high ecological relevance that contextually replicates the train illusion compared to a VE with low ecological relevance that depicts optic flow. We hypothesized that participants would be more likely to experience vection in a VE with high ecological relevance that contextually replicates the train illusion compared to a VE with low ecological relevance that depicts optic flow, which would be operationalized by higher self-reported feedback measures.

#### 1.3.2. Sensory modality

We aimed to compare whether vection is enhanced by the addition of auditory feedback to visual feedback in VEs with low and high ecological relevance. We hypothesized that participants would be more likely to experience vection when auditory feedback was added to visual feedback compared to applying visual feedback alone, which would be operationalized by higher self-reported feedback measures.

#### 1.3.3. Physiological responses

We aimed to identify whether the experience of vection elicited an activation of the sympathetic nervous system. We hypothesized that when participants experienced vection, an activation of the sympathetic nervous system would occur. The activation of the sympathetic nervous system would be characterized by (i) mydriasis of the pupil owing to the relaxation of the ciliary muscle, (ii) increased cardiac output and heart rate (HR) owing to an increased cardiac innervation, (iii) bronchodilation which may increase breathing rate (BR) and/or the depth of breath, and (iv) increased EDA owing to sweat gland activation [75–77]. Thus, we expected that the increased cardiovascular, electrodermal, pupillary and respiratory activity would correlate to a quicker, more intense and convincing, and a longer vection experience.

#### 1.3.4. Understanding participants' vection experience

We aimed to identify how participants compared their experience of vection in environments with different levels of ecological relevance to the description of the train illusion. We hypothesized that participants' vection experience of the train illusion in real life would be more relatable to their vection experience in a VE replicating the train illusion compared to their vection experience in a VE depicting optic flow. The higher relatability between vection experiences in real life and the VE would be operationalized by higher ratings of relatability to the description of the train illusion. Lastly, we aimed to explore how participants experienced vection in real life and VR through open-ended questions.

## 2. Method

This study was approved by the Deakin University Human Research Ethics Committee (DUHREC, 2021-181). Written informed consent was obtained from the participants at the start of the experiment, and no incentives (e.g. money or course credits) were offered to them.

### 2.1. Sample

A total of 29 (*F* = 9, *M* = 20) participants with a mean age of 30.4 (s.d. = 5.1, min = 23, max = 42) years volunteered for this study. Participants were recruited from the staff and student body of the Institute for Intelligent Systems Research and Innovation at Deakin University as well as through snowball sampling. Twenty participants reported to not have a refractive error, seven participants reported myopia (min = −2.75, max = −0.5), one participant reported mild hyperopia (left eye = +0.25, right eye = +0.25), and one participant reported mild anisometropia (left eye = +0.25, right eye = −0.75). None of the participants were excluded based on their refractive errors.

### 2.2. Materials

**Figure 1.**
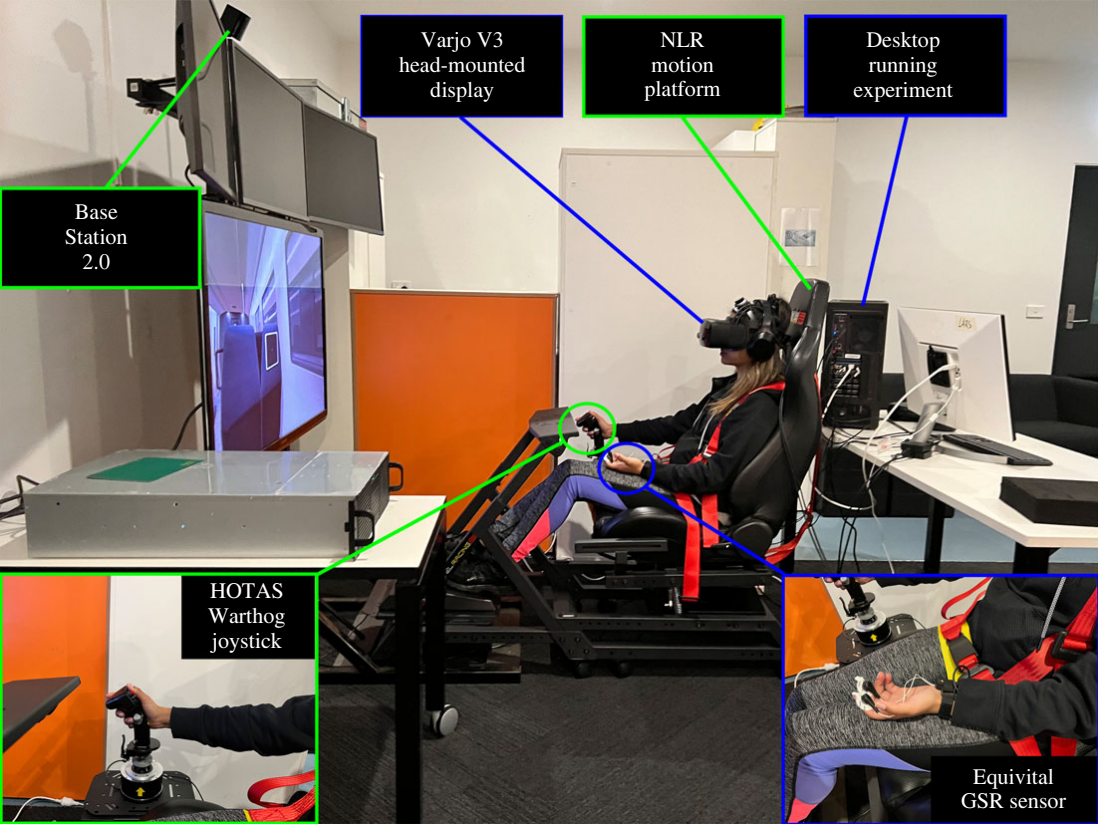
The experimental set-up used in this study. Note: a participant is shown who is seated in the NLR Motion Platform, wearing the Varjo V3 head-mounted display and the Equivital galvanic skin response (GSR) sensor while using a HOTAS joystick to indicate their vection experience. The Equivital EQ02 is worn underneath the clothing.

Participants were immersed in a custom-designed VE developed in Unity 2019.4.f21. A desktop computer running a 64-bit version of Windows 10 with Intel(R) Core(TM) i9-10900K CPU @ 3.70 GHz, NVIDIA GeForce RTX 3090 24GB graphics card, Z490 AORUS ELITE MOBO and 64GB RAM was used to run the VE and record participants' pupillary activity and joystick input. The participants sat on the NLR Motion Platform V3, were visually immersed using the Varjo V3 head-mounted display (HMD), and audibly immersed using Samsung WH-1000XM4 closed-back and noise-cancelling headphones. The HMD was run using Varjo Base software (v.3.4.1.10), Varjo XR Plugin (v.2.3.0) and Unity XR plugin (v.4.2.1), tracked by a single SteamVR Base Station (v.2.0), and contained an integrated eye tracker that sampled at 200 Hz. Moreover, the participants were outfitted with a small harness containing the Equivital EQ02, which was used to record a variety of participants’ physiological signals. The Equivital records body accelerations in microgravity and ECG in millivolts at 256 Hz, respiratory waveforms in arbitrary units at 25.6 Hz and EDA in millisievert at 16 Hz. Furthermore, participants wore an auxiliary sensor on their hands to record EDA. Finally, participants used a Thrustmaster HOTAS Warthog Flight Stick to provide feedback during stimulus presentation. The experimental set-up is shown in figure 1.

### 2.3. Design

The research design was within-subjects and was composed of three independent variables (IVs) where each IV had two levels. The IVs were variations to the VEs in which participants were immersed. Participants were visually and audibly immersed in either a VE depicting a train carriage or a VE showing a point cloud using the HMD and noise-cancelling headphones. In the environments, the participants were asked to indicate their experience of vection using a joystick for the duration of the trial. During the first 10 s of each trial, the participants were acclimatized to the environment and objects remained stationary. Subsequently, objects in the environment accelerated linearly over 10 s, after which they moved at a constant velocity for 60 s. After 60 s of constant velocity, the participants were switched to a new environment in which they answered a set of questions. The total duration of each trial was 80 s. The overall experimental procedure lasted about 1 h and 15 min.

### 2.4. Independent variables

The first IV was the *feedback modality*, which comprised two levels: (i) visual, and (ii) visual and auditory. Visual stimuli were presented via the HMD, and auditory stimulation was presented to the participant through closed-back and noise-cancelling headphones.

**Figure 2.**
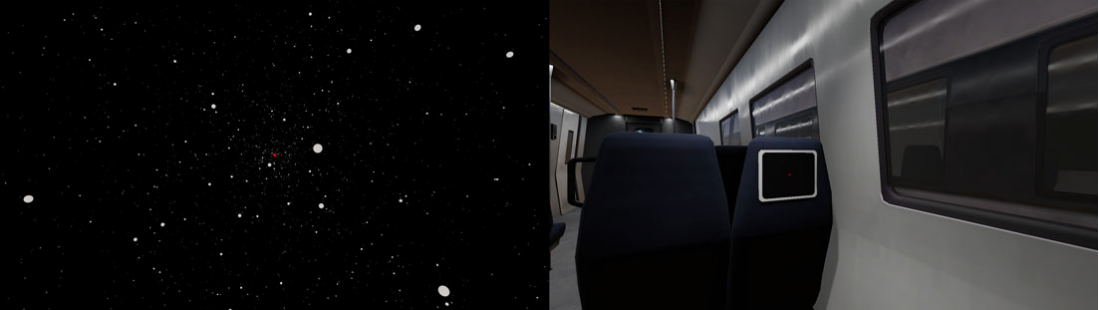
The virtual environments used in this study. Note: left: an optic flow condition which was used as an abstract motion stimulus. During this stimulus, a white point cloud moved towards the observer. Right: the train condition which aimed to contextually replicate the train illusion. During this stimulus a moving train could be seen through the window on participants' right-hand side. The windows on the left were blinded.

The second IV was *the ecological relevance* of feedback type, which was either abstract or naturalistic. The abstract visual stimulus consisted of a moving point cloud simulating optic flow (figure 2, left). We chose to use optic flow patterns as these patterns are the most commonly used visual stimulation type in vection research (e.g. [53,55]). Furthermore, optic flow patterns provide ambiguous motion information of the spheres (i.e. is the observer moving towards the spheres or are the spheres moving towards the observer) in a similar, although contextually limited, fashion as the trains do in the train illusion (i.e. is the train of the observer moving or is the adjacent train moving). Researchers either use optic flow patterns with a limited lifetime (e.g. [78,79]) or add/relocate points once existing points hit a virtual boundary (e.g. [53]). We decided to simulate 5000 points moving at 15 m s^−1^ with a clipping plane at 780 m and to relocate points that moved outside the bounding area of a virtual sphere, akin to the ‘slow’ condition in the study by Keshavarz *et al*. [53]. Even though the slow condition (i.e. 15 m s^−1^) elicited weaker vection than the fast condition (i.e. 75 m s^−1^) in the study by Keshavarz *et al*. [53], we chose the slow velocity to make the optic flow condition comparable to the (naturalistic) train condition. Conversely put, it would appear extremely unrealistic to have a train accelerate to 270 km h^−1^ (i.e. 75 m s^−1^) within 10 s if we implemented the velocity of the fast condition used in the study by Keshavarz *et al*. [53]. The abstract auditory stimulus was an ascending Shepard–Risset Glissando (SRG), as previous research on auditory vection has shown that the SRG elicits vection [64,80]. We obtained a Shepard–Risset audio sample from the same source^1^ used by Mursic *et al*. [64]. The naturalistic visual stimulus depicted the participant seated in a train carriage adjacent to another train (figure 2, right). The adjacent train received a velocity of 15 m s^−1^ (i.e. 54 km h^−1^), which is similar to the velocity achieved in the optic flow condition. The naturalistic auditory stimulus was the sound of the train departing from a station in Bielefeld, Germany.

The third IV was whether participants had to perform a *secondary task* or not. Thus, participants were either expected to perform no secondary task or a visual discrimination reaction time task (VDRTT). For trials in which participants did not have to perform a secondary task, they were instructed to just retain their focus on a red marker at the far depth of their view (figure 2). Visual focus markers have been employed widely in vection research (e.g. [26,81–84]). During trials where participants were expected to perform the VDRTT, the participants were required to press a button on the joystick as fast as possible when they saw the colour of the focus marker change. The details and results of the VDRTT and the comparison to the trials wherein participants did not have to perform a secondary task have been reported elsewhere [85]. Herein, we have only reported the results of the trials where participants did not perform a secondary task.

In summary, participants were presented with a unique randomized order of the eight (two sensory modalities × two ecological relevance × two secondary task) conditions. The virtual conditions lasted in total about (8 × 80 s)/60 s = 11 min.

### 2.5. Procedure

The experimental procedure was conducted in line with COVID-19 restrictions. Participants were welcomed by the first author as the experimenter and were informed about the goal of the experiment, namely, to ‘*investigate if and how humans experience movements in virtual environments*'. The participants completed a COVID-19 screening form, were asked to read a plain language statement, and signed a consent form. After reading the plain language statement and signing the consent form, a questionnaire containing questions about demographics, current well-being, and a shortened version of Bett's Questionnaire on Mental Imagery [86] was administered (for results, see [87]). After completing the questionnaire, participants were shown how to attach the Equivital harness to themselves and retreated to a private room where they could attach the device underneath their clothing. Upon return, the experimenter attached the auxiliary electrodermal sensor to the left hand of the participant, and participants subsequently sat on the motion platform. After fastening the seatbelt of the motion platform, the participants were instructed how to use the HMD and joystick. After familiarization with the equipment, the participants put on the HMD and the position of the HMD was calibrated. The simulator was launched, and a practice trial was initiated in which participants were familiarized with the tasks of the experiment. Previous research has shown that task instruction can affect vection [58]. Therefore, we refrained from verbally giving instructions or feedback to the participants and presented written task instructions that were not suggestive of the nature of the stimuli in order to avoid instilling a potential bias in participants regarding (i) the concept of vection, and (ii) the context of the train illusion. The task instructions for the practice round and the actual experiment are detailed in the electronic supplementary material and were adapted from the instructions denoted in the studies conducted by Palmisano *et al*. [55] and Palmisano & Chan [58]. Prior to the start of the practice trial, a 5-point calibration was used to calibrate the eye tracker.

The practice trial consisted of a point cloud of 5000 points moving at 10 m s^−1^ and rotating at 5 deg s^−1^, similar to the practice condition of Keshavarz *et al*. [53]. After the practice trial, the participants were asked to rate statements measuring vection intensity, vection convincingness, perceived self-motion velocity, presence and CS. The first four statements were rated using the visual analogue scale (VAS). The statements for vection intensity and convincingness were ‘*Please rate how strong your feeling of movement was*’ and ‘*Please rate how convincing your feeling of movement was*’. The statement for self-motion velocity was ‘*Please rate how fast you were going*’. The wording of the intensity, convincingness, and self-motion statements was derived from the recommendations detailed by Soave *et al*. [88] who investigated the terminology used in vection research. We adapted the recommendation to include a vection intensity and convincingness rating (e.g. [29,37,89]). The left and right anchors of the vection intensity and convincingness ratings were ‘*not at all*’ and ‘*extremely intense*’, and ‘*not at all*’ and ‘*extremely convincing*’. The marker to rate these statements was placed in the middle of the VAS line. The perceived self-motion velocity rating did not have a left anchor. The right anchor displayed the integer speed in km h^−1^ that participants rated they perceived they were going. The marker was placed on the left side of the VAS line (i.e. participants moved the joystick to the right to increase the velocity value). Furthermore, participants indicated their virtual presence by rating the statement ‘*In the computer generated world I had a sense of “being there”*’, which was the first question from the questionnaire developed by Slater & Usoh [90]. Participants used a VAS line with the left and right anchors being ‘*not at all*’ and ‘*very much*’, respectively, to rate this statement. The marker was placed in the middle of the VAS line. Lastly, participants rated CS by means of the motion illness symptoms classification (MISC; [91]; originally the MIsery Scale; [92–94]) ranging from 0 to 10, where 0 represents no problems, 1 represents asymptomatic uneasiness, 2 and 3 represent vague to slight discomfort, and 4 and greater indicate more severe symptoms of discomfort. After completing these questions, a dark screen was presented, and participants were verbally asked by the experimenter if they had any questions. If the participants indicated that they understood the course of the experiment, they were handed noise-cancelling headphones. After putting on the headphones, the experiment was initiated and another 5-point calibration was performed to calibrate the eye tracker. If participants decided to take a break and took the headset off during the experiment, the calibration was re-performed. After calibration, participants were presented with one of the two instructions specific to the experimental condition through the HMD (see the electronic supplementary material). The participants could progress through the experiment at their own pace by pressing the fire button on the joystick.

The same procedure was repeated for each unique combination of IVs. The presentation order of the IVs was randomized for each participant. Participants were free to take a break after they submitted their responses to the questions (i.e. before the start of the next stimulus presentation), and they were reminded that they had the option to pause after the four stimuli. After the completion of all combinations of IVs, the participants were instructed to remove the HMD. The experimenter assisted the participants and offered them water. Participants then took off the Equivital harness in private and, once they indicated that they were ready to proceed, a post-experiment questionnaire was administered.

The questionnaire contained a description of the train illusion, as detailed by James [49], and participants were asked whether they had previously experienced vection in real life. Participants were provided with the option, if they had experienced vection, to disclose *where* they had the experience. Participants were then presented with an image showing the optic flow environment, as depicted in figure 2, and asked the following questions. ‘*Consider the description by William James. How does William James's description relate to your experience in the environment depicted above?*' which participants rated using a VAS line with left and right anchors ‘*Does not relate at all*' and ‘*is completely relatable*’. Participants were asked to elaborate on their ratings for a minimum of 10 words. Subsequently, participants were asked, ‘*How would you explain your motion experience in the environment above to someone who is unfamiliar with VR?*’ to which they could type their answer. After answering these questions, the same process was repeated underneath the depiction of the train environment. Upon submitting the questionnaire, the participants were thanked for their participation and provided the opportunity to ask questions about the experiment.

### 2.6. Data reduction and analysis

The joystick position data, questionnaire responses, pupil size data recorded via the eye tracker in the Varjo HMD, and physiological data recorded via the Equivital were read in a custom MATLAB script (see the Data accessibility statement for a link to the script and data) and time-series data were synchronized. All data reduction and analysis were performed using MATLAB. The data reduction steps and the computation of dependent variables are detailed per data source. Dependent variables were computed for every trial, and a trial consisted of a 10 s acclimation time, 10 s of linear acceleration time and 60 s of constant velocity.

#### 2.6.1. Joystick data

Joystick position data were linearly interpolated to achieve a constant sampling frequency of 100 Hz and were then filtered using a moving average filter with a window size of (100 Hz/60 s) two samples. From the joystick data, participants' vection experience (VExp) was derived by taking the root of the squared sum of the horizontal and vertical joystick positions $\left(i.e.\;\mathrm{VExp}=\sqrt{{\mathrm{joystick}}_{\mathrm{vertical}}^{2}+{\mathrm{joystick}}_{\mathrm{horizontal}}^{2}}\right)$. From VExp, the following chronometric dependent variables were computed.

1. Vection onset time (VOT; in seconds): the difference between the moment the trial started (*t*_trial,start_) and the moment VExp exceeded $0(t_{\mathrm{VExp}>0})$ [37]:
  2.1 $$\mathrm{VOT}=\;t_{\mathrm{VExp}>0}-t_{\mathrm{trial},\mathrm{start}}.$$

If the participant did not move the joystick during the trial, a VOT of 80 s was used.

2. Vection duration (VD; in seconds): the sum of the difference between the moments VExp exceeded $0(t_{\mathrm{VExp}>0})$ and reached 0 again $\left(t_{\mathrm{VExp}=0}\right)$:
  2.2 $$\mathrm{VD}=\overset{n}{\underset{i=1}{\sum }}t_{\mathrm{VExp}=0,i}-t_{\mathrm{VExp}>0,i}.$$

A vection percentage was computed for each condition based on the number of participants from the total sample who indicated that they experienced vection by moving the joystick. In summary, a combination of five vection measures were recorded to capture the different aspects of vection [20].

#### 2.6.2. Eye tracker data

Missing eye data owing to blinks were linearly interpolated [95] and data were subsequently linearly interpolated to 400 Hz to achieve a constant sampling frequency. The Varjo V3 outputted the pupil diameters (PDs) in arbitrary units ranging from 0 to 1, which we multiplied by 100 to better reflect the 0 to 100% range that the eye tracker could measure. PD samples were then filtered using a zero-delay third-order low-pass Butterworth filter with a cut-off frequency of 4 Hz [96]. Subsequently, mean PD in arbitrary units was computed by computing the average PD over the duration of the trial for each participant.

#### 2.6.3. Equivital data

The data recorded by the Equivital were exported into separate files per physiological signal and each physiological signal was cleaned independently. The following manipulation steps were performed per signal.

##### 2.6.3.1. Cardiovascular activity

Data of cardiovascular activity, recorded through an ECG, were cropped to the duration of each trial. Data were then band-pass filtered using a zero-delay fourth-order Butterworth filter with a low-pass cut-off frequency of 40 Hz and a high-pass cut-off frequency of 0.5 Hz [97]. From the filtered ECG signal, the HR was obtained. HR in beats per minute was computed by counting the peaks of the QRS complex, dividing the number of peaks by the duration of the trial, and multiplying it by 60 s, for each participant independently.

##### 2.6.3.2. Electrodermal activity

The EDA samples were cropped over the total duration of the experiment, z-scored, and subjected to a convex optimization approach using the default settings of cvxEDA except for $\tau_{0}$ which was optimized between 2.0 s and 4.0 s for each participant independently [98]. cvxEDA was used to separate the phasic and tonic component of EDA. Subsequently, the obtained phasic and tonic signals were cropped to the duration of each trial and the mean EDA for the phasic (EDA_p_) and tonic (EDA_t_) components in arbitrary units (arb. units) was computed by taking the average of these signals over the total duration of a trial (i.e. 80 s) for each participant independently.

##### 2.6.3.3. Physical activity

Data of participants’ body movements, recorded through the accelerometer, were cropped to the duration of each trial. Data were then band-pass filtered using a zero-delay fourth-order Butterworth filter with a low-pass cut-off frequency of 11 Hz and a high-pass cut-off frequency of 0.5 Hz [99]. Physical activity (PA in microgravity) was derived by computing the Euclidian norm of the triaxial accelerometer signals and taking the average of the signal over the duration of the trial for each participant independently.

##### 2.6.3.4. Respiratory activity

Respiratory waveforms were cropped to the duration of each trial and linearly interpolated to 51 Hz to achieve a constant sampling frequency. Subsequently, the data were band-pass filtered between 4 and 60 breaths per minute [100], reflecting a low-pass cut-off frequency of 1 Hz and a high-pass cut-off frequency of 1/15 Hz, using a third-order zero-delay Butterworth filter. BR in breaths per minute were computed using the advanced counting method (ACM) proposed by Schäfer & Kratky [101] using the code provided by Charlton *et al*. [100]. The ACM went as follows: the filtered respiratory signal was band-pass filtered again between 0.1 and 0.5 Hz using a third-order Butterworth filter from which the local maxima and minima were derived. Subsequently, the vertical differences between the maxima and minima were determined from which the third quantile was derived. The third quantile was multiplied by 0.3 to obtain a threshold value which was used to find pairs of subsequent extrema in the respiratory waveform. The vertical distances between extrema which surpassed the threshold were classified as a breathing cycle. Once all pairs of extrema had been evaluated, the average BR was computed by dividing 60 s by the mean length of all the breathing cycles.

#### 2.6.4. Statistical comparisons

Outliers of the physiological dependent variables were removed using the method of Rousseeuw & Croux [102], which has been shown to be a robust method of removing outliers in psychophysiological data (see [103]). Criticism in the scientific literature on the use of the *p*-value in null-hypothesis testing as an indicator of (non)significant effects has highlighted the need to interpret results in a non-dichotomous way [104,105]. To assist the readership in interpreting differences in the data, within-subject compatibility intervals (i.e. formerly known as 95% confidence intervals) are reported for all dependent variables alongside point estimates and standard deviations (e.g. see [106]). These intervals were derived using the method detailed by Morey [107] in which the mean score of all conditions per participant was subtracted for each variable when computing 95% confidence intervals. Amrhein & Greenland [108, p. 316] denoted that compatibility intervals encompass ‘*… the possible effect sizes that are most compatible with the data …, given the assumptions*.' In other words, the compatibility intervals will portrait the range of plausible values surrounding the mean value that are compatible with our recorded data based on our statistical assumptions. Additionally, conventional inferential statistical comparisons were performed at the participant level. The dependent variables were subjected to two-way repeated-measures ANOVAs to assess the effect of ecological relevance and sensory modality. In the event the assumptions for the ANOVA were not met, the Scheirer–Ray–Hare test was used [109,110]. In addition, we performed Spearman's rank correlations between the dependent variables. The alpha level was set at 0.05.

## 3. Results

Some of the results detailed in this article have been published as conference proceedings. An analysis was performed to investigate the influence of kinaesthetic imagery on vection [87]. Furthermore, a correlational analysis was performed on the dependent variables vection intensity, presence and CS to investigate the influence of CS on the relationship between vection and presence [111]. Finally, the effect of a secondary task on vection has been reported [85].

Owing to a technical error, some of the responses of eight participants to the questions in the post-experiment survey related to the naturalistic environment were not retained; therefore, the post-experiment survey data of these participants were not included in the analysis. Nonetheless, the data for all other dependent measures reported in the Results section contain all 29 participants (unless specified otherwise). However, the data and analysis of the dependent measures for the 21 participants for whom the post-experiment survey data were retained are reported separately in the electronic supplementary material.

### 3.1. Vection

Based on the joystick responses, 82.8% of the participants reported vection in the abstract visual condition, whereas 72.4% of the participants reported vection in the abstract visual–audio condition. In the naturalistic visual condition, vection was reported by 62.1% of the participants, and 58.6% of the participants reported vection in the naturalistic visual–audio condition.

**Figure 3.**
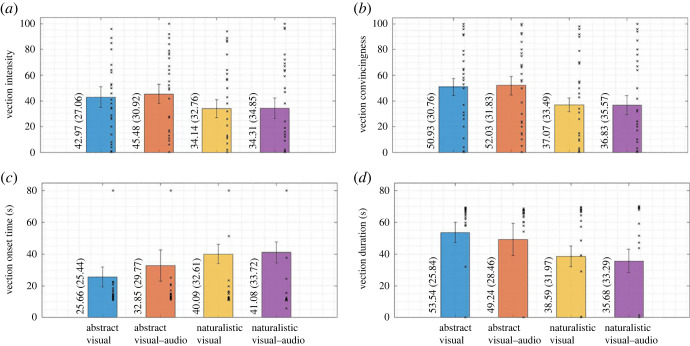
Mean, standard deviation and compatibility interval of vection measures. Note: means (and standard deviations in parentheses) are denoted numerically for each condition in the appropriate bar. Individual data points are marked with ‘*x*'. Error bars represent the compatibility intervals.

Figure 3 details the mean, standard deviation and compatibility interval of each vection measure. Vection intensity was, on average, higher in the abstract conditions compared to the naturalistic conditions. However, the partially overlapping compatibility intervals between the abstract and naturalistic condition suggest that the differences were not compatible with the rejection of our first hypothesis. Similarly, no substantial differences were found between visual and visual–audio conditions as can be seen from the fully overlapping compatibility intervals. A two-way repeated-measures ANOVA corroborated the aforementioned notions as it showed no main effects of ecological relevance (*F*_1,28_ = 2.51, *p* = 0.124, $\eta_{p}^{2}=0.08$) or sensory modality (*F*_1,28_ = 0.33, *p* = 0.568, $\eta_{p}^{2}=0.01$). Lastly, no interaction effect was found (*F*_1,28_ = 0.45, *p* = 0.507, $\eta_{p}^{2}=0.02$).

Vection convincingness was, on average, rated higher in abstract conditions compared to the naturalistic conditions. The non-overlapping compatibility intervals suggest that the differences are compatible with the rejection of the first hypothesis. Furthermore, no substantial differences were found between the visual and visual–audio conditions, as can be seen from the fully overlapping compatibility intervals. A two-way repeated-measures ANOVA corroborated the potential rejection of our first hypothesis as a main effect for ecological relevance was found (*F*_1,28_ = 7.60, *p* = 0.010, $\eta_{p}^{2}=0.21$). Additionally, no effect of sensory modality (*F*_1,28_ = 0.03, *p* = 0.865, $\eta_{p}^{2}<0.01$) nor an interaction effect (*F*_1,28_ = 0.16, *p* = 0.688, $\eta_{p}^{2}=0.01$) was found.

On average, vection occurred later in the naturalistic conditions than in the abstract conditions, as can be seen from the higher VOTs. VOTs were the smallest in the abstract visual condition compared to the other conditions; however, the partially overlapping compatibility intervals suggest that the differences were not compatible with the rejection of our first hypothesis. Similarly, no substantial differences between the visual and visual–audio conditions were observed, as can be seen from the overlapping compatibility intervals. A Scheirer–Ray–Hare test found no main effects (ecological relevance: *H* < 0.01, *p* = 0.975, *E*^2^ < 0.01; sensory modality: *H* = 0.13, *p* = 0.716, *E*^2^ < 0.01) nor an interaction effect (*H* = 1.70, *p* = 0.192, *E*^2^ = 0.06).

On average, vection lasted longer in the abstract conditions than in the naturalistic conditions; however, only the differences between the abstract visual and naturalistic visual–audio condition appeared to be compatible with the rejection of our hypothesis, as indicated by the non-overlapping compatibility intervals. Differences between the visual and visual–audio conditions were not substantial, as can be seen from the overlapping compatibility intervals. A Scheirer–Ray–Hare test revealed no main effects (ecological relevance: *H* = 0.57, *p* = 0.449, *E*^2^ = 0.02, sensory modality *H* = 0.25, *p* = 0.617, *E*^2^ = 0.01) nor an interaction effect (*H* = 1.39, *p* = 0.238, *E*^2^ = 0.05).

The means, standard deviations and compatibility intervals for the 21 participants from which the post-experiment survey data were obtained are shown in the electronic supplementary material, table S1. Furthermore, electronic supplementary material, table S2 details the results of the two-way repeated-measures ANOVA on the vection measures of these participants. A similar large, although non-significant, effect of ecological relevance on vection convincingness was observed.

### 3.2. Perceived self-motion velocity

On average, participants rated their perceived self-motion velocity to be lower in the abstract visual (*M* = 20.52, s.d. = 21.51, compatibility interval (CI) = [14.64 26.40]) and visual–audio (*M* = 24.55, s.d. = 26.17, CI = [19.18, 29.92]) conditions compared to the naturalistic visual (*M* = 32.03, s.d. = 30.22, CI = [25.79, 38.28]) and visual–audio (*M* = 25.24, s.d. = 27.62, CI = [18.12, 32.36]) conditions. Furthermore, perceived self-motion ratings were, on average, higher for the abstract visual–audio condition compared to the abstract visual condition, whereas perceived self-motion velocity ratings were, on average, higher for the naturalistic visual condition compared to the naturalistic visual–audio condition. Nonetheless, the (partially) overlapping compatibility intervals suggests that the differences were not substantial. A Scheirer–Ray–Hare test confirmed the absence of substantial differences between conditions as no main effects (ecological relevance: *H* = 0.07, *p* = 0.792, *E*^2^ < 0.01, sensory modality *H* = 0.19, *p* = 0.663, *E*^2^ = 0.01) nor an interaction effect (*H* = 0.96, *p* = 0.328, *E*^2^ = 0.03) were found.

### 3.3. Presence and cybersickness

Presence was rated, on average, higher in the abstract visual (*M* = 54.38, s.d. = 27.61, CI = [48.67, 60.09]) condition than in the naturalistic visual condition (*M* = 50.34, s.d. = 31.68, CI = [44.22, 56.47]), whereas presence ratings were, on average, lower in the abstract visual–audio (*M* = 51.59, s.d. = 32.54, CI = [45.53, 57.64]) condition than in the naturalistic visual–audio (*M* = 55.59, s.d. = 29.19, CI = [49.97, 61.20]) condition. Nonetheless, the overlapping compatibility intervals between abstract and naturalistic conditions as well as between visual and visual–audio conditions suggest that the differences were not substantial. A two-way repeated-measures ANOVA found no main effects of ecological relevance (*F*_1,28_ < 0.01, *p* = 0.997, $\eta_{p}^{2}<0.01$) or sensory modality (*F*_1,28_ = 0.25, *p* = 0.618, $\eta_{p}^{2}=0.01$). However, an interaction effect was found (*F*_1,28_ = 5.87, *p* = 0.022, $\eta_{p}^{2}=0.17$), suggesting that the addition of auditory cues to the abstract condition lowered presence, whereas adding sound to the naturalistic condition increased presence ratings. A similar interaction effect was found for the subset of 21 participants, as shown in the electronic supplementary material, table S3.

**Figure 4.**
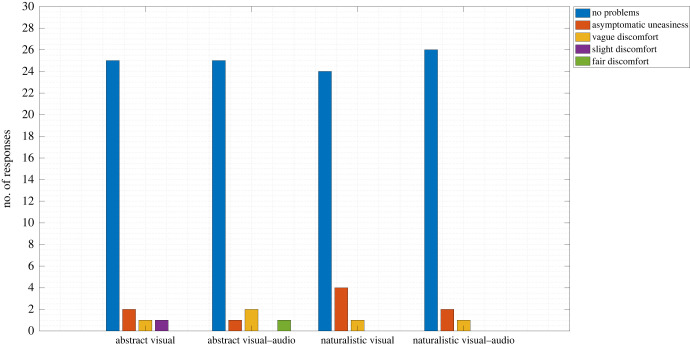
Distribution of participants' MISC ratings.

Twenty-two participants reported no discomfort throughout the experiment, whereas four reported asymptomatic uneasiness as their worst symptom. Two participants reported vague symptoms of discomfort and one participant reported strong symptoms of discomfort. Figure 4 depicts the distribution of the MISC ratings for each condition. More participants reported discomfort in the naturalistic visual condition; however, the strongest discomfort symptom was reported in the abstract visual–audio condition. A Scheirer–Ray–Hare test revealed no main effects (ecological relevance: *H* = 0.02, *p* = 0.897, *E*^2^ < 0.01, sensory modality *H* = 0.22, *p* = 0.642, *E*^2^ = 0.01) nor an interaction effect (*H* = 0.29, *p* = 0.590, *E*^2^ = 0.01). However, when MISC ratings for each condition were compared to the null option of ‘no symptoms’, the naturalistic visual condition appeared to be the most uncomfortable condition for participants (see the electronic supplementary material, table S4).

### 3.4. Physiological responses

Table 1 details the mean, standard deviation and compatibility interval of the physiological signals. Additionally, the table denotes the results of statistical comparisons between experimental conditions for the physiological signals. The results for each of the physiological signals are discussed in the following sections.

**Table 1.** Mean, standard deviation and compatibility interval of the physiological signals and results of statistical comparisons between experimental conditions. (Note: arb. units, arbitrary units; bpm, beats per minute; BR, breathing rate; brpm, breaths per minute; CI, compatibility interval; EDA_p_, average phasic electrodermal activity; EDA_t_, average tonic electrodermal activity; HR, heart rate, *M*, mean; mG, microgravity; *n*, number of participants retained after outlier removal, PA, physical activity; PD, pupil diameter, s.d. standard deviation. *p*-values below the alpha level are indicated in bold font.)

	abstract visual				abstract visual–audio				naturalistic visual				naturalistic visual–audio			
variable	*M*	s.d.	CI	*n*	*M*	s.d.	CI	*n*	*M*	s.d.	CI	*n*	*M*	s.d.	CI	*n*
BR (brpm)	17.18	1.66	16.83,17.53	26	18.23	1.38	17.81,18.65	28	16.88	2.26	16.44,17.33	29	17.87	2.31	17.29,18.44	28
EDA_p_ (arb. units)	0.08	0.08	0.07,0.10	22	0.05	0.05	0.03,0.07	19	0.11	0.10	0.09,0.13	26	0.09	0.08	0.07,0.10	21
EDA_t_ (arb. units)	−0.12	0.60	−0.35,0.11	25	−0.23	0.73	−0.50,0.04	28	−0.70	0.69	−0.98,−0.43	26	−0.12	0.67	−0.35,0.12	26
HR (bpm)	70.03	10.06	69.19,70.86	29	70.75	9.14	69.73,71.78	29	70.16	9.92	69.28,71.03	29	70.70	10.41	69.90,71.50	29
PA (mG)	2.03	0.29	1.97,2.09	27	2.12	0.41	2.04,2.20	27	2.17	0.43	2.06,2.29	27	2.22	0.41	2.12,2.33	29
PD (arb. units)	7.38	1.13	7.24,7.51	28	7.57	1.32	7.42,7.71	29	7.55	1.29	7.39,7.71	29	7.84	1.30	7.73,7.95	29
			*ecological relevance*					*sensory modality*					*interaction*			
BR (brpm)			*H* = 0.70, *p* = 0.402, *E*^2^ = 0.03					*H* = 6.77, ***p* = 0.009**, *E^2^* = 0.24					*H* = 0.37, *p* = 0.545, *E^2^* = 0.01			
EDA_p_ (arb. units)			*H* = 0.66, *p* = 0.418, *E*^2^ = 0.02					*H* = 0.55, *p* = 0.457, *E^2^* = 0.02					*H* = 0.08, *p* = 0.780, *E^2^* < 0.01			
EDA_t_ (arb. units)			*H* = 1.67, *p* = 0.196, *E*^2^ = 0.06					*H* = 2.12, *p* = 0.146, *E^2^* = 0.08					*H* = 6.46, ***p* = 0.011**, *E^2^* = 0.23			
HR (bpm)			*F*_1,28_ = 0.01, *p* = 0.929, $\eta_{p}^{2}<0.01$					*F*_1,28_ = 1.43, *p* = 0.241, $\eta_{p}^{2}=0.05$					*F*_1,28_ = 0.05, *p* = 0.826, $\eta_{p}^{2}<0.01$			
PA (mG)			*F*_1,26_ = 4.76, ***p* = 0.038**, $\eta_{p}^{2}=0.15$					*F*_1,26_ = 0.53, *p* = 0.472, $\eta_{p}^{2}=0.02$					*F*_1,26_ = 1.00, *p* = 0.327, $\eta_{p}^{2}=0.04$			
PD (arb. units)			*F*_1,27_ = 2.58, *p* = 0.120, $\eta_{p}^{2}=0.09$					*F*_1,27_ = 14.88, ***p* = 0.001**, $\eta_{p}^{2}=0.36$					*F*_1,27_ = 2.17, *p* = 0.152, $\eta_{p}^{2}=0.07$			

#### 3.4.1. Breathing rate

On average, BR was higher in the abstract compared to the naturalistic conditions; however, the (partially) overlapping compatibility intervals suggest that the differences were not substantial. Additionally, BR was, on average, higher in the visual–audio conditions compared to the visual-only conditions. However, only the difference between visual and visual–audio for the abstract condition was substantial, as can be seen from the non-overlapping compatibility intervals, whereas the compatibility intervals in the naturalistic condition depict a marginal, partial overlap. A Scheirer–Ray–Hare test confirmed an effect of sensory modality on BRs (table 1) and showed no main effect of ecological relevance nor an interaction effect.

#### 3.4.2. Electrodermal activity

Phasic EDA was, on average, higher in the naturalistic conditions compared to the abstract conditions, however, the overlapping compatibility intervals suggest the differences were not substantial. Participants exhibited, on average, higher phasic EDA in the visual-only conditions compared to the visual–audio conditions; however, these differences were not substantial as indicative of the overlapping compatibility intervals. A Scheirer–Ray–Hare test also showed no main or interaction effects (table 1).

Tonic EDA was, on average, lower in the naturalistic visual condition compared to the abstract visual condition, however, tonic activity was lower in the abstract visual–audio condition compared to the naturalistic visual–audio condition. A substantial difference between the naturalistic and abstract visual condition as well as between the naturalistic visual and visual–audio condition can be identified by the non-overlapping compatibility intervals, whereas differences between the abstract visual and visual–audio condition were not substantial as indicated by the (partially) overlapping compatibility intervals. A Scheirer–Ray–Hare test found no main effects but did identify an interaction effect (table 1).

#### 3.4.3. Heart rate

HR was, on average, higher in the naturalistic compared to the abstract visual condition; however, it was, on average, lower in the naturalistic compared to the abstract visual–audio condition. Nonetheless, the overlapping compatibility intervals suggest that the differences were not substantial. On average, participants exhibited a higher HR in the visual–audio conditions compared to the visual-only conditions; however, the overlapping compatibility intervals also indicate that the differences were not substantial. A two-way repeated-measures ANOVA corroborated the lack of differences as no main or interaction effects were identified (table 1).

#### 3.4.4. Physical activity

Physical activity was, on average, higher in the naturalistic condition compared to the abstract condition; however, the partially overlapping compatibility intervals suggest that these differences were not substantial. Similarly, physical activity was, on average, higher in the visual–audio conditions compared to the visual-only conditions, however, the partially overlapping compatibility intervals indicate that these differences were not substantial. Nonetheless, a two-way repeated-measures ANOVA identified a main effect for ecological relevance but not for sensory modality nor an interaction effect (table 1).

#### 3.4.5. Pupil diameter

PD was, on average, smaller for abstract conditions compared to their naturalistic counterpart; however, the partially overlapping compatibility intervals suggest that the differences were not compatible with the confirmation of our first hypothesis. Furthermore, PD was, on average, larger for visual–audio conditions compared to visual conditions, however, only the differences for the naturalistic condition were substantial, as is indicative from the non-overlapping compatibility intervals. A two-way repeated-measures ANOVA showed a main effect of sensory modality, but not for ecological relevance nor any interaction effect (table 1).

#### 3.4.6. Correlations to vection measures

Tables 2–5 detail Spearman rank correlations between the vection measures and physiological measures for each condition. The physiological measures presented weak to moderate, non-significant correlations to vection measures across the four conditions, which does not provide a basis for the acceptance of our third hypothesis, except for a moderate negative correlation between tonic EDA and vection intensity (*r* = −0.50) in the abstract visual condition and a moderate positive correlation between PD and VD (*r* = 0.43) in the naturalistic visual condition.

**Table 2.** Spearman rank-order correlation matrix between dependent variables for the abstract visual condition. (Note: mean, standard deviation and compatibility interval for dependent measures can be found in figure 3, tables 1 and 2. arb. units., arbitrary units; bpm, beats per minute; brpm, breaths per minute; mG, microgravity; MISC, motion illness symptoms classification. Asterisks indicate the significance of *p*-values. *p < 0.05, **p < 0.01 and ***p < 0.001.)

	1	2	3	4	5	6	7	8	9	10	11	12
1. vection intensity												
2. vection convincingness	0.93***											
3. vection onset time	−0.60*	−0.72**										
4. vection duration	0.60*	0.72**	−1.00***									
5. velocity	0.67**	0.74***	−0.60*	0.60*								
6. presence	0.89***	0.95***	−0.65**	0.65**	0.70**							
7. MISC	−0.14	−0.11	−0.27	0.27	−0.17	−0.16						
8. breathing rate (brpm)	0.21	0.23	−0.01	0.01	0.16	0.29	−0.21					
9. electrodermal activity, phasic (arb. units)	−0.38	−0.34	0.29	−0.29	−0.23	−0.37	0.28	0.05				
10. electrodermal activity, tonic (arb. units)	−0.50*	−0.42	0.22	−0.22	−0.27	−0.42	−0.05	0.03	0.43			
11. heart rate (bpm)	0.20	0.16	−0.22	0.22	0.04	0.11	0.37	−0.49*	0.00	−0.30		
12. physical activity (mG)	−0.35	−0.26	0.13	−0.13	0.06	−0.29	−0.08	0.24	−0.01	0.33	−0.30	
13. pupil diameter (arb. units)	−0.08	−0.24	0.00	0.00	0.11	−0.16	0.13	−0.03	0.02	0.35	−0.20	0.18

**Table 3.** Spearman rank-order correlation matrix between dependent variables for the abstract visual–audio condition. (Note: mean, standard deviation, and compatibility interval for dependent measures can be found in figure 3, tables 1 and 2. arb. units, arbitrary units; bpm, beats per minute; brpm, breaths per minute; mG, microgravity; MISC, motion illness symptoms classification. Asterisks indicate the significance of *p*-values. *p < 0.05, **p < 0.01 and ***p < 0.001.)

	1	2	3	4	5	6	7	8	9	10	11	12
1. vection intensity												
2. vection convincingness	0.72**											
3. vection onset time	−0.36	−0.17										
4. vection duration	0.31	0.11	−0.93***									
5. velocity	0.74**	0.73**	−0.34	0.31								
6. presence	0.46	0.65**	0.14	−0.28	0.44							
7. MISC	0.34	0.17	−0.36	0.40	−0.01	−0.22						
8. breathing rate (brpm)	0.27	0.11	0.27	−0.17	−0.04	0.45	0.09					
9. electrodermal activity, phasic (arb. units)	−0.04	0.31	−0.11	0.02	0.23	0.27	−0.31	−0.17				
10. electrodermal activity, tonic (arb. units)	0.05	−0.12	0.05	−0.12	−0.11	−0.25	0.17	−0.40	−0.30			
11. heart rate (bpm)	0.28	0.43	−0.29	0.17	0.08	0.14	0.43	0.02	−0.03	0.19		
12. physical activity (mG)	0.37	0.06	−0.19	0.10	0.50*	0.19	−0.26	−0.17	0.15	−0.17	−0.47	
13. pupil diameter (arb. units)	0.03	0.05	−0.16	0.30	0.29	−0.13	−0.04	−0.14	−0.17	0.11	−0.18	0.19

**Table 4.** Spearman rank-order correlation matrix between dependent variables for the naturalistic visual condition. (Note: mean, standard deviation and compatibility interval for dependent measures can be found in figure 3, tables 1 and 2. arb. units, arbitrary units; bpm, beats per minute; brpm, breaths per minute; mG, microgravity; MISC, motion illness symptoms classification. Asterisks indicate the significance of *p*-values. *p < 0.05 and ***p < 0.001.)

	1	2	3	4	5	6	7	8	9	10	11	12
1. vection intensity												
2. vection convincingness	0.98***											
3. vection onset time	−0.75***	−0.81***										
4. vection duration	0.73***	0.79***	−0.97***									
5. velocity	0.72***	0.76***	−0.83***	0.80***								
6. presence	0.40	0.41	−0.26	0.32	0.16							
7. MISC	0.24	0.16	−0.10	0.00	0.09	0.00						
8. breathing rate (brpm)	0.07	0.02	0.22	−0.27	−0.34	0.10	0.09					
9. electrodermal activity, phasic (arb. units)	−0.09	−0.15	0.15	−0.13	−0.20	−0.06	0.11	0.00				
10. electrodermal activity, tonic (arb. units)	0.12	0.24	−0.16	0.11	0.08	0.19	0.18	−0.08	0.06			
11. heart rate (bpm)	0.22	0.25	−0.21	0.24	0.22	0.01	0.26	0.01	0.12	0.35		
12. physical activity (mG)	0.16	0.17	0.00	0.02	−0.10	0.14	−0.05	0.41	−0.03	0.00	−0.30	
13. pupil diameter (arb. units)	0.07	0.11	−0.33	0.43*	0.14	0.44*	0.01	−0.24	−0.06	0.13	0.22	−0.15

**Table 5.** Spearman rank-order correlation matrix between dependent variables for the naturalistic visual–audio condition. (Note: mean, standard deviation and compatibility interval for dependent measures can be found in figure 3, tables 1 and 2. arb. units, arbitrary units; bpm, beats per minute; brpm, breaths per minute; mG, microgravity; MISC, motion illness symptoms classification. Asterisks indicate the significance of *p*-values. *p < 0.05, **p < 0.01 and ***p < 0.001.)

	1	2	3	4	5	6	7	8	9	10	11	12
1. vection intensity												
2. vection convincingness	0.98***											
3. vection onset time	−0.87***	−0.86***										
4. vection duration	0.87***	0.89***	−0.99***									
5. velocity	0.62**	0.66**	−0.61**	0.64**								
6. presence	0.39	0.38	−0.27	0.24	0.23							
7. MISC	0.21	0.24	−0.35	0.35	0.42	0.04						
8. breathing rate (brpm)	0.39	0.38	−0.18	0.21	−0.01	0.13	0.05					
9. electrodermal activity, phasic (arb. units)	−0.21	−0.25	0.11	−0.14	−0.13	0.08	0.06	−0.51*				
10. electrodermal activity, tonic (arb. units)	−0.02	−0.06	0.20	−0.23	−0.14	0.13	−0.55*	−0.16	0.32			
11. heart rate (bpm)	0.34	0.30	−0.40	0.37	0.21	−0.13	−0.11	−0.19	−0.04	0.05		
12. physical activity (mG)	−0.12	−0.09	0.16	−0.16	−0.13	0.15	−0.43	−0.04	0.20	0.52*	−0.48*	
13. pupil diameter (arb. units)	0.26	0.27	−0.15	0.23	0.28	0.24	−0.02	0.28	−0.16	−0.02	−0.28	0.12

### 3.5. Post-experiment survey

Relatability to James' description for the naturalistic environment (*M* = 61.43, s.d. = 35.54, *n* = 21) was rated, on average, higher than the ratings for the abstract environment (*M* = 52.67, s.d. = 32.10, *n* = 21); however, a right-tailed *t*-test showed that the ratings for the naturalistic environment were not significantly larger than the ratings for the abstract environment (*t*_20_ = 1.41, *p* = 0.087). Furthermore, a Spearman rank correlation between the relatability ratings for the abstract and naturalistic conditions showed a positive relationship between the two ratings (*r*_19_ = 0.59, *p* = 0.005), which suggests that participants were likely to provide similar ratings of relatability to William James' description of vection for both environments. The reliability rating for the abstract condition exhibited non-significant, small to moderate correlations with vection measures for the abstract visual and abstract visual–audio conditions, except for a moderate negative correlation between VOT in the abstract visual condition (*r*_19_ = −0.47, *p* = 0.033), as can be seen in the electronic supplementary material, table S5. Conversely, relatability ratings for the naturalistic condition correlated strongly with vection intensity, vection convincingness, VOT, and VD in the naturalistic visual and visual–audio conditions, as can be seen in the electronic supplementary material, table S6.

Seventeen participants indicated that they had experienced vection in real life, three participants were unsure, and one participant indicated that they had never experienced vection in real life before. Thirteen participants had experienced vection while seated on a train; however, some participants reported that they had also experienced vection at other locations, such as on a boat or in a car. One participant indicated that they experienced a feeling of movement when they watched waves at sea, whereas another reported having experienced vection while seated on a plane before taking off. Six participants indicated that they had experienced vection while seated in a car. The experience of vection in a car was described by participant 20 to occur ‘*… when stopping at* [a] *red light but cars from sides still move slowly*’. Similarly, Participants 15 and 29 noted the occurrence of vection when ‘*… my car was not moving*[,] *and the other car started moving*' and ‘*…someone stop*[s] *beside me and they move,* [I] *feel my car is moving*'.

Based on the elaboration of the participants' experience in the abstract environment, ten participants described that they experienced ‘pure' vection. For example, participant 11 detailed ‘*I feel that I was moving toward all the* [objects] *around me*', whereas participant 17 denoted ‘[t]*his indeed felt like I was moving mainly because I have never experienced a similar environment before*'. Four participants experienced a mixture of vection and object-motion. For example, participant 19 noted that ‘*… it felt like objects were moving towards me unless I concentrated and willed myself to think I was moving*'. This participant also noted an explicit contextual difference between the optic flow environment and the train illusion, stating that in the optic flow conditions there were ‘*… no windows and there* [were] *many small objects rather than one big object (train) which was moving, then stopped, then started moving again*'. Four participants described that they only experienced the objects in the environment moving towards them, which describes object-motion. Seven participants described their experiences in the abstract environment as space-like, similar to participants in the study by Guterman & Alison [112]. For example, participant 9 described ‘… *I* [felt like I was] *floating in space towards the red dot …*', whereas participant 11 detailed ‘*… you feel like you are in real space and you* [travel] *from one* [galaxy] *to another one*'.

Overall, fewer participants described the experience of vection in the naturalistic environment. Based on the feedback, only six participants described the experience of ‘pure' vection. For example, participant 12 described that ‘*… it was very* [realistic] *and you get to have an experience* [similar] *to real world.* [I]*f not identical.*' Similarly, participant 25 denoted it felt ‘… [l]*ike being on a train and feeling as though I'm moving but I wasn't*’. By contrast, eight participants detailed accounts of object-motion. For example, participant 17 described ‘*… I always felt like the train next to me was moving.* [M]*ainly because I did* [not] *feel the vibration from my train*'. Six participants detailed the experience of a mixture of vection and object-motion, and they noted ambiguity in the directionality of motion in the VE. For example, participant 23 described that they ‘*… could not differentiate* [whether] *the train* [was] *moving forward or the other train* [was] *moving at some point*'. Similarly, participant 28 noted: ‘*I assumed my train was moving but I* [had] *no stationary reference point to base that off*'. Some participants highlighted the involvement of attention in their vection experience. For example, participant 27 described: ‘*I felt motion more when I was only vaguely paying attention.* [F]*ocusing harder on the coloured spot decreased the feeling of motion*'. Conversely, participant 22 detailed ‘*… while focusing on the red dot … I felt the train heading forward*'. Participant 16 described that: ‘*I didn't feel like I was moving when focusing on the dot. However,* [i]*f I looked outside I felt a sense of movement*'.

## 4. Discussion

Herein, we aimed to examine whether vection is experienced differently depending on the ecological relevance of the stimulus and identify how participants compare their experience of vection in environments with different levels of ecological relevance to the description of the train illusion. Twenty-nine participants were visually and audibly immersed in VEs, depicting either optic flow or a moving train carriage. Participants provided real-time feedback on their vection experience using a joystick in each trial and *post hoc* quantitative feedback on their vection experience after each trial. Furthermore, a post-experiment survey was administered to record qualitative feedback on participants' vection experience. The aims of our study were subdivided into four sections, namely (i) ecological relevance, (ii) sensory modality, (iii) physiological responses, and (iv) understanding participants' vection experience. In the following sections our findings are discussed in more detail.

### 4.1. The effect of ecological relevance of a stimulus on vection

We expected that participants would be more likely to experience vection in a VE that contextually replicated the train illusion than in a VE that depicted optic flow. In contrast to previous research [14,59], we found that more convincing vection was elicited by a stimulus with low ecological relevance (i.e. optic flow) than by a stimulus with high ecological relevance (i.e. the train environment), which provides basis to reject our first hypothesis. Our findings can be explained, in part, by the dynamic content of the visual stimuli. The dynamic content of the stimuli can roughly be subdivided into three components, namely the (i) field of view, (ii) spatial distribution (i.e. density) of motion cues, and (iii) perceived velocity of motion cues. Firstly, in the naturalistic scene, the dynamic content was limited to the area visible through the window of the train carriage, whereas whole field-of-view stimulation was present in the abstract condition. The difference in field-of-view may have caused the naturalistic conditions to be less effective in eliciting vection compared to the abstract conditions. For example, Allison *et al.* [113] found that larger degrees in field of view had a positive effect on vection ratings in participants who sat in a tumbling room. Similarly, Keshavarz *et al*. [26] found that vection ratings were higher when participants viewed vertical gratings on a dome projection subtending a larger field of view compared to viewing gratings on a single screen or a flat projection screen. Furthermore, the presence of a resting foreground in the naturalistic condition may have inhibited vection as well; even though it has been shown that motion cues behind a stationary foreground can facilitate vection (e.g. [114,115]), the presence of a foreground in the naturalistic condition may have had a negative effect on the genesis of vection compared to the abstract condition that did not have a foreground. It is worth noting that one participant (P16) declared in the post-experiment survey that they did not experience vection when they focused on the marker on the back of the seat, but they did when they looked out of the window and one participant (P27) noted that increasing their focus on the red dot on the back of the seat decreased their vection experience. However, two other participants (P11, P22) described positive vection experiences while having their focus on the marker on the back of the seat. Although focus markers can assist in mitigating CS [116], their use and placement should be carefully considered in relation to the dynamic content of the stimuli. Nonetheless, owing to the field of view restriction by the stationary foreground, the density of the stimulus also differed between the abstract and the naturalistic condition. Density in this context means the tally of objects or particles per unit area [117]. The effect of stimulus density can be highlighted by the findings in the study by Keshavarz *et al*. [53] where the authors found that optic flow patterns with a higher stimulus density (i.e. 5000 points) elicited stronger vection than patterns with a lower stimilus density (i.e. 200 points). Sequentially, this may have affected the perceived velocity of the stimuli as Guo *et al*. [118] found that when moving horizontal gratings had a higher density, these stimuli were not only perceived to be moving faster by participants, but they also elicited stronger vection compared to horizontal gratings with lower density. Thus, even though the ecological relevance of the stimulus does play a role in the genesis and modulation of vection [14], it appears to play less of a role compared to the dynamic content of the stimuli.

### 4.2. The effect of bi-modal stimulation on vection

Based on previous findings in literature [4,7,9], we hypothesized that participants' vection experience would be enhanced by the addition of auditory feedback to visual feedback in VEs with low and high ecological relevance. By contrast, we found that the addition of auditory cues did not substantially increase participants' vection experience. Although the *post hoc* reports on vection intensity and convincingness seem to suggest the addition of sound *increased* participants' vection experience, our results for the temporal vection measures seem to suggest that the addition of sound *decreased* participants' vection experience, more notably so in the abstract condition compared to the naturalistic condition. For the abstract condition, our findings can, in part, be explained by the possibility that the illusory increase in pitch that the SRG aims to induce may have reduced vection in the abstract condition as it may be more compatible with upward vection compared to the forward vection that the optic flow pattern aimed to elicit. The participant reports from Mursic *et al*. [64] showed that upwards vection was reported more frequently in response to the SRG compared to forward vection. The potential conflict between the upward vection induced by the SRG and the forward vection induced by the optic flow pattern in our experiment may have been tempered by the fact that our stimulus incorporated a section of linear acceleration, to which the SRG may have been compatible to (see [119] on the use of the SRG for acceleration sounds). The slight differences in the naturalistic condition may have been a result from a potential incompatibility between the simulated velocity of the *sound* of the moving train and the visuals of the moving train, which potentially could explain the lower average perceived self-motion velocity rating in the naturalistic visual–audio condition compared to the naturalistic visual condition. However, none of the participants reported a discrepancy between the perceived velocities of the audio and visual feedback in the naturalistic condition in the post-experiment questionnaire.

### 4.3. Physiological responses to vection

We expected that when participants experienced vection an activation of the sympathetic nervous system would occur, which would present itself through pupil dilation, increased HR, increased BR, and increased EDA as a correlate to a quicker, more intense, convincing and prolonged vection experience. Our results from the Spearman rank correlations showed that almost none of the physiological indices correlated to the vection measures, and thus our results do not support a confirmation of our third hypothesis.

Furthermore, the experimental manipulations did not appear to affect most of the physiological measures; however, some influences were found for pupillary and physical activity measures. Specifically, the ANOVAs showed an effect of sensory modality on PD, which suggests that PDs were larger when visual and auditory feedback was presented compared to conditions with visual feedback only. This dilative effect was most prominent between the naturalistic conditions. The larger pupil dilation in response to bi-modal stimuli can, in part, be explained by multisensory integration [120,121]. For example, in the study by Wang *et al*. [121] participants performed saccadic eye movements in response to visual, audio or audio–visual cues while their pupil size and gaze behaviours were recorded. The authors found an additive effect from bi-modal stimulation on participants' pupil size compared to unimodal audio or visual stimulation. However, the dilative effect of the addition of auditory stimulation appears to be most prominent for the naturalistic condition opposed to the abstract condition. As such, it alternatively may be that the dilation in response to the auditory feedback was owing to uncertainty; since the auditory clip initiated with the sound of an idling engine, participants might have started integrating this sensory information to try and decide which of the two trains had started their engine (see [122], for a review on dilative effects of auditory stimulation). Results from the ANOVAs on physical activity measures indicated an effect of ecological relevance on PA, indicating that participants were more active during the naturalistic conditions compared to the abstract conditions. This effect can, in part, be explained by the visual context of the naturalistic environment; participants were shown to be seated on the train and they might have looked around the train or shifted their position during the acclimatization condition, which may have resulted in the higher average physical activity found for those conditions compared to the abstract conditions in which participants were merely shown a dark environment with white optical points dispersed.

### 4.4. The context of the train illusion

We hypothesized that participants' vection experience in a VE replicating the train illusion would be more relatable to the vection experience of the train illusion in real life than their vection experience in a VE depicting optic flow. We found that the relatability ratings for the train illusion did not differ between the abstract and naturalistic environments; however, the relatability rating for the naturalistic environment correlated well with the self-reported vection measures for these environments. The results of our study did not confirm or reject our fourth hypothesis. The relatability ratings to William James' description of the train illusion did not differ between the naturalistic and abstract environments, which suggests that the description was equally comparable to either environment. However, the Spearman rank correlations showed that participants' reported vection experience in naturalistic environments aimed to replicate the train illusion correlated well to the relatability rating for this environment. Moreover, the self-reported vection measures for the abstract environment did not correlate well with the relatability ratings for this environment, suggesting that the description of the train illusion may not be fully representative of participants' vection experience in abstract environments. The post-experiment survey also revealed a discrepancy between the self-reported vection measures, and the experience detailed by the participants. From the joystick data detailed in the electronic supplementary material, two-thirds of participants indicated they experienced vection in the naturalistic condition, however, only half of the participants described their movement experience in the naturalistic condition with terminology related to feelings of movement (e.g. participant 15 ‘*I* [felt] *my train was moving*’). The opposite was noted for the abstract condition; the joystick data reported in the electronic supplementary material show that more than 80% of participants experienced vection in the abstract visual condition and over two-thirds of participants indicated that they experienced vection in the abstract visual–audio condition. In the post-experiment survey 70% of the participants used terms such as ‘feeling' to describe their experience of self-movement in the abstract environment (e.g. participant 16 ‘*I felt I was moving slowly …*'). The discrepancy regarding the feedback for the naturalistic environment can, in part, be explained by the type of stimulus used in the practice round. The visual cue was a helically moving point cloud which was contextually akin to the abstract condition. As such, the participants may have been more familiar with the sensations in the abstract conditions compared to the naturalistic conditions. Alternatively, it may be that participants responded to the *perception* of self-motion instead of the *feeling* of self-motion in the naturalistic environment, as they saw the adjacent train moving and thus inferred that it was either themselves or the train moving. For example, participant 24 detailed that they questioned whether the other train was moving, and they were stationary: ‘*I … visually swapped the perceived motion to be my own. But I concluded … that it was the other train that was moving and I was stationary*'. In summary, vection research would highly benefit from an interview study wherein participants' lived accounts of vection were assessed to formulate appropriate task instructions and self-reported measures using the correct terminology (e.g. [88]). Alternatively, researchers should consider open-ended post-experiment questionnaires where participants can detail their experience which can be used to accurately interpret the self-reported measures.

### 4.5. Limitations, implications and outlook

As one of the few works in the literature to complement their quantitative vection experiment with a qualitative survey, our study has a few limitations. First, our study was limited by the small sample size. A *post hoc* power calculation (MorePower; [123]) using the main effects of ecological relevance for the vection measures (i.e. $\eta_{p}^{2}=0.08-0.21$) revealed that our current test power ranged from 33% to 74%. Conversely, to adequately detect large effects $\left(\eta_{p}^{2}=0.14\right)$, an additional 23 participants needed to be recruited to achieve 80% test power. Second, our use of a joystick instead of a button to record VOT may have been a limiting factor in this measure, as participants may have extended their arms without inclining the joystick. Nonetheless, our results theoretically imply that the elicitation of vection is not solely a bottom-up exercise wherein stimulus characteristics play a leading role, but that vection is subjected to higher-order cognitive influences, and that participants' self-reporting on vection may be volatile to heuristics.

Future studies could further investigate the effect of stimulus context by presenting participants with a variety of VEs where the dynamic content of each environment is matched to one another. *A priori* test could be conducted where participants rate the perceived velocity of the stimuli to ensure that the dynamic content is objectively and subjectively matched. Additionally, a comparison between two groups could be made where one group is instructed about vection using the TIA and one group is instructed using a ‘neutral' description of vection in VEs.

## 5. Conclusion

This study investigated whether vection was experienced differently depending on the ecological relevance of the VE used to elicit vection and explored how participants' vection experience in these environments related to the context of the train illusion through open-ended questions in a post-experiment survey. Based on our findings and previous studies in the vection literature, it is concluded that the ecological relevance of the stimulus may play less of a role than the dynamic content of the stimulus in the elicitation of vection. However, higher-order cognitive influences on self-reported vection measures should always be considered as discrepancies were found between participants' quantitative vection measures and qualitative reports. As such, more qualitative future research is needed to understand participants' vection reporting behaviour and to determine the appropriate terminology to formulate participants' task instructions and robust vection measures.

## Data Availability

The data and source code are accessible via: https://doi.org/10.26187/deakin.22281313 [124] and the supplementary materials are linked via reference [125].

## Footnotes

1 Audio file sampled from https://soundcloud.com/shepard-tone.
